# Risk stratification in intermediate asymptomatic carotid artery disease: a multi-modal approach combining local flow dynamics and systemic clinical profiles

**DOI:** 10.3389/fsurg.2026.1872735

**Published:** 2026-08-28

**Authors:** Panagiotis Siogkas, Dimitrios Pleouras, Vassiliki Potsika, Vassilis Tsakanikas, Fragkiska Sigala, George Galyfos, George Charalampopoulos, Igor Koncar, Dimitrios I. Fotiadis

**Affiliations:** 1 Dept. of Materials Science and Engineering, University of Ioannina, Ioannina, Greece; 2 First Propedeutic Department of Surgery, National and Kapodistrian University of Athens, Athens, Greece; 3 Department of Vascular and Endovascular Surgery, Faculty of Medicine, University of Belgrade, Belgrade, Serbia

**Keywords:** cardiovascular risk stratification, carotid artery stenosis, computational fluid dynamics, hemodynamics, machine learning, stenosis progression, wall shear stress

## Abstract

**Background/objectives:**

Carotid artery stenosis is a leading cause of ischemic stroke, yet accurate patient-specific prediction of its progression remains an unresolved clinical challenge. Neither baseline stenosis severity nor conventional cardiovascular risk factors can reliably identify which asymptomatic patients will progress. This study aimed to develop and validate a machine learning (ML) framework for predicting carotid stenosis progression by integrating patient-specific computational fluid dynamics (CFD) features with clinical and imaging data.

**Methods:**

A dataset of 146 carotid arteries from 129 asymptomatic patients enrolled in the multi-center TAXINOMISIS project was used. Stenosis progression was defined as a > 10% increase at the latest available follow-up assessment (56/146 arteries, 38.4%). Twenty-two features were derived from clinical risk factors and CFD-based hemodynamic metrics including time-averaged wall shear stress (TAWSS), oscillatory shear index (OSI), relative residence time (RRT), and disturbed flow indices. ML classifiers were trained using patient-level GroupShuffleSplit, Optuna Bayesian hyperparameter optimization (200 trials), patient-level 5-fold GroupKFold cross-validation, 1,000-iteration bootstrap confidence intervals, and calibration analysis. Performance was benchmarked against two clinically motivated baselines: baseline stenosis grade alone and clinical risk factors alone.

**Results:**

The proposed pipeline achieves substantially better discrimination than either clinical baseline: the best-performing model (RF + XGBoost Ensemble, Optuna-tuned) reached AUC=0.814 (95% CI: 0.702–0.917; Sensitivity=0.609; Specificity=0.828; Brier score=0.184), versus AUC=0.552 for stenosis grade alone and AUC=0.459 for clinical risk factors alone — neither of which provides meaningful discrimination. The combined stenosis + clinical model (AUC=0.388) also failed to improve upon either comparator, confirming that hemodynamic information is the necessary added value. Feature contribution analysis consistently identified the normalized area of low TAWSS (Low TAWSS %) as the dominant predictor across all classifiers, followed by the disturbed flow area (LOHA) and vessel-average OSI.

**Conclusions:**

A multimodal, physics-informed ML pipeline integrating patient-specific CFD features substantially outperforms all clinical benchmarks for predicting carotid stenosis progression. These findings demonstrate that hemodynamic phenotyping is necessary to advance beyond current risk stratification approaches in asymptomatic carotid disease and support the development of CFD-integrated ML as a clinical decision-support tool in vascular surgery.

## 1 Introduction

The application of artificial intelligence (AI) in vascular surgery has paved the way for advanced diagnostics and patient-specific risk stratification. Systematic reviews encompassing over 200 ML studies in vascular surgery have demonstrated a median AUROC of 0.88 across applications ranging from diagnosis to prognosis and image segmentation (1–3), and ML models have proven particularly effective in analyzing complex, high-dimensional datasets that combine imaging and clinical information to predict outcomes which are difficult to characterize via conventional statistical approaches (4).

Carotid artery disease represents one of the most compelling cases for this approach, as personalizing risk could fundamentally change the way we prevent strokes. Driven primarily by atherosclerosis, the progressive narrowing of the carotid arteries substantially increases the risk of ischemic stroke—a leading cause of global mortality and long-term disability. The TAXINOMISIS clinical trial, a prospective multi-center European study, was specifically designed to address the inadequacy of current risk stratification models for asymptomatic carotid artery disease, recognizing that existing approaches—based primarily on stenosis degree and conventional cardiovascular risk factors—have shown limited ability to identify which individual patients will progress (5–7). This limitation has direct clinical consequences: many strokes arise from asymptomatic arteries that do not meet the traditional criteria for high-risk intervention. The threshold for intervention remains controversial, largely because evidence from population-level trials cannot reliably predict an individual's prognosis (6, 8). This gap makes tailored prevention difficult to achieve in practice.

Computational fluid dynamics (CFD) has long been applied to the carotid bifurcation to characterize the hemodynamic environment governing plaque development and progression. Foundational work by Hyun et al. (9), Filipovic et al. (10), Marshall et al. (11), and Morbiducci et al. (12) established that time-averaged wall shear stress (TAWSS) and the oscillatory shear index (OSI) are strongly associated with regions prone to plaque formation and rupture. Subsequent refinements in boundary condition methodology and patient-specific geometry reconstruction (13–15) demonstrated that CFD can accurately replicate intravascular dynamics. The roles of low and oscillatory WSS—and composite indices such as relative residence time (RRT), normalized WSS (nWSS), WSS heterogeneity index (WHI), and the disturbed flow area (LOHA)—in driving endothelial dysfunction and atherogenesis are now well-established (16–20). Patient-specific hemodynamic simulation has also been explored in the context of carotid atherosclerosis natural history (21, 22) and post-interventional remodeling, underscoring the mechanistic primacy of flow environment over static anatomical measurements.

In parallel, the field has shifted toward predictive modeling. Statistical and ML approaches have been used to identify individuals at high risk for significant stenosis (23–25, 45, 48, 49) and to predict progression of carotid intima-media thickness (26–28, 46). More recent studies have applied deep learning to CT angiography (CTA) for symptomatic plaque detection (29–31, 47) and AI models to electronic health records for clinical decision support (32). ML has also demonstrated strong performance in predicting outcomes following carotid endarterectomy and transcarotid artery revascularization (33–35). However, these approaches are largely limited to imaging or clinical data alone and do not incorporate the biophysical forces governing plaque growth and progression.

A critical frontier is the integration of CFD with ML—a paradigm increasingly referred to as physics-informed ML. By embedding mechanistic knowledge of hemodynamics directly into the feature space, this approach goes beyond purely data-driven prediction: it leverages the known causal pathways of atherogenesis as inductive biases that guide the model toward physically meaningful solutions. Traditional hemodynamic simulations, while mechanistically informative, have been considered too computationally intensive for routine clinical prognosis (36). Recent works using deep learning surrogates for CFD (37–39) have begun to bridge this gap. Our group previously demonstrated the feasibility of this integrated approach in a preliminary study on 87 carotid arteries, where an XGBoost model trained on CFD features achieved an AUC of 0.741 (40, 50). The present study substantially extends that work by expanding the dataset to 146 arteries, enriching the feature set with novel hemodynamic descriptors (RRT, nWSS, WHI, LOHA), applying a rigorous validation framework, and formally benchmarking against two clinically meaningful comparators: baseline stenosis grade alone—the metric currently guiding treatment decisions—and conventional clinical risk factors alone.

The present study aims to demonstrate that a multimodal ML framework integrating patient-specific CFD features substantially outperforms both clinically available baselines for predicting stenosis progression, and to identify which hemodynamic features drive this improvement.

## 2 Materials and methods

### 2.1 Dataset

This study included 146 carotid arteries from 129 patients with asymptomatic moderate-to-severe carotid stenosis enrolled in the prospective multi-center TAXINOMISIS project (ClinicalTrials.gov: NCT03495830) (5). Participating centers included NKUA (Athens, Greece), UMC (Utrecht, Netherlands), UBEO (Belgrade, Serbia), and USMI (Milan, Italy). All subjects provided written informed consent; the study was approved by local institutional ethics review boards at each center in accordance with the Declaration of Helsinki. “Asymptomatic” was defined as the absence of ipsilateral TIA, amaurosis fugax, or ischemic stroke within the preceding 6 months. Exclusion criteria included prior ipsilateral CEA or CAS, post-radiation stenosis, carotid dissection, inflammatory vasculopathy, life expectancy <2 years, and inability to provide written informed consent.

Baseline high-resolution black-blood MRI was acquired on a a) 1.5-Tesla GE Signa HDx system with a bilateral four-channel phased-array carotid coil (NKUA), b) 3-Tesla Philips Medical Systems Achieva with a bilateral four-channel phased-array carotid coil (UMC), c) 3-Tesla SIEMENS Skyra system with a bilateral four-channel phased-array carotid coil (UBEO), d) 3-Tesla SIEMENS Prisma with a bilateral four-channel phased-array carotid coil (USMI), respectively. Follow-up duplex ultrasound assessments were performed at 1, 2, and 3 years post-enrollment. An artery was classified as a progressor (Class 1) if its NASCET stenosis degree had increased by >10% absolute at the latest completed follow-up assessment relative to baseline; otherwise it was classified as stable (Class 0). Of 146 arteries, 56 (38.4%) met the progression criterion (Class 1) and 90 (61.6%) remained stable (Class 0). Table 1 presents baseline demographics. The TAXINOMISIS protocol enrolled patients with at least one carotid artery with ≥50% stenosis by NASCET (patient-level criterion); 104/146 arteries (71.2%) met this threshold, while the remaining 42 (28.8%) are contralateral arteries of bilateral patients under active vascular surveillance. For each of the 146 included carotid arteries, the CFD-derived hemodynamic features, eCRF clinical parameters, and progression outcome label were linked via a unique patient-artery identifier, ensuring strict one-to-one correspondence across all three data streams. The ToF-MRI images, duplex ultrasound measurements, and eCRF clinical records are all derived from the same 129 patients — there is no mixing of data from different patient populations.

**Table 1:** Dataset demographics description.

Characteristic	N (%)
Patients (n)	129
Age, years (mean ± SD)	69.4 ± 8.4
Male sex	84 (65.1%)
Carotid arteries included	146
Progression (Class 1, >10% increase)	56 (38.4%)
Stable (Class 0)	90 (61.6%)
Stenosis Grade 1 (0%–30%)	13 (8.9%) contralateral arteries
Stenosis Grade 2 (30%–50%)	29 (19.9%) contralateral arteries
Stenosis Grade 3 (50%–70%)	56 (38.4%) qualifying arteries
Stenosis Grade 4 (70%–100%)	48 (32.9%) qualifying arteries
Smoking (Current + Former)	90 (70.1%)
Diabetes mellitus	45 (34.9%)
Hypertension	107 (83.0%)
Hypercholesterolemia	100 (77.4%)
Coronary artery disease	34 (26.4%)
BMI (mean ± SD)	26.48 ± 3.4

Patient-specific carotid geometries were reconstructed from Time-of-Flight (ToF) MRI using an in-house deep learning pipeline (41): three U-Net architectures trained on 485 annotated images from 42 subjects were used to segment the lumen across the CCA, ICA, and ECA. Following segmentation, 3D level-set morphological refinement and marching cubes surface meshing were applied. Transient blood flow simulations were performed in ANSYS CFX solving the incompressible Navier–Stokes equations (Equations 1, 2):

$$\rho (\partial u/\partial t\;+\;u\cdot \nabla u)\;=\;-\nabla \;p\;+\;\mu \nabla^{2}u\;,$$ [1]

∇⋅u=0, [2]

where u is the velocity vector (m/s), p is pressure (Pa), *ρ* is blood density and μ is dynamic viscosity. Blood was modeled as a Newtonian fluid (*ρ*=1,050 kg/m^3^, μ=0.0035 Pa·s). The Newtonian approximation is appropriate for the physiological shear rate range in stenotic carotid arteries, where non-Newtonian effects on TAWSS and OSI are typically below 5%–8% (42). Patient-specific inlet mass flow rate profiles were derived from duplex ultrasound PSV and EDV measurements spanning at least three cardiac cycles, using an in-house developed MATLAB script. Mean CCA flow rates across the cohort ranged from 4.2 to 9.8 mL/s, consistent with published reference values for carotid blood flow (11). A zero-pressure boundary condition was applied at the ICA outlet; no-slip, no-penetration conditions were enforced at arterial walls. The implemented mesh consisted entirely of tetrahedra (maximum face element size 0.16 mm), optimized after a mesh sensitivity analysis comparing discretizations of 0.32 mm, 0.16 mm, and 0.08 mm; TAWSS values converged to within 3% between the 0.16 mm and 0.08 mm meshes, confirming mesh-independent results for the adopted discretization (convergence criterion 10⁻⁴, ≤150 iterations per time step). Mean arterial pressure was computed as MAP=(SBP+2·DBP)/3 and pressure ratios P_ICA_/P_CCA_ and P_ECA_/P_CCA_ were derived from simulation outputs.

### 2.2 Feature extraction and labeling

Twenty-two features were extracted per artery across three domains: clinical risk factors, imaging/morphological parameters, and CFD-derived hemodynamic metrics. The complete feature set is presented in Table 2. Clinical features included artery topology, smoking status, diabetes presence and type, hypertension, coronary artery disease, hypercholesterolemia, mean arterial pressure, baseline stenosis grade (coded 1–4 corresponding to 0%–30%, 30%–50%, 50%–70%, and 70%–100% stenosis, derived from multi-center eCRF data), and a composite Clinical Risk Burden (CRB) score (0 = no risk factors, 5 = all five risk factors present).

**Table 2:** Complete feature set used for model training, organized by domain.

Domain	Feature	Unit/Scale
Clinical	Artery side	Binary (0 = Left, 1 = Right)
Clinical	Smoker	Ordinal (1 = Current, 2 = Former, 3 = Never)
Clinical	Diabetes	Ordinal (1 = TypeI, 2 = TypeII, 3 = No)
Clinical	Hypertension	Binary (1 = Yes, 2 = No)
Clinical	Coronary artery disease	Binary (1 = Yes, 2 = No)
Clinical	Hypercholesterolemia	Binary (1 = Yes, 2 = No)
Clinical	Mean arterial pressure (MAP)	mmHg
Clinical	Baseline stenosis grade	Ordinal (1–4)
Clinical	Clinical Risk Burden (CRB)	Composite score
Hemodynamic (CFD)	Peak TAWSS	Pa
Hemodynamic (CFD)	Vessel-average TAWSS	Pa
Hemodynamic (CFD)	Vessel-average OSI	0–0.5
Hemodynamic (CFD)	Area of low TAWSS	m^2^
Hemodynamic (CFD)	Low TAWSS area/Total lumen area	%
Hemodynamic (CFD)	Area of high OSI	m^2^
Hemodynamic (CFD)	High OSI area/Total lumen area	%
Hemodynamic (CFD)	PICA/PCCA	Ratio
Hemodynamic (CFD)	PECA/PCCA	Ratio
Hemodynamic (CFD)	Relative Residence Time (RRT)	Pa⁻^1^
Hemodynamic (CFD)	Normalized WSS (nWSS)	Dimensionless
Hemodynamic (CFD)	WSS Heterogeneity Index (WHI)	Dimensionless
Hemodynamic (CFD)	Disturbed Flow Area (LOHA)	Dimensionless

TAWSS, time-averaged wall shear stress; OSI, oscillatory shear index; RRT, relative residence time; nWSS, normalized WSS; WHI, WSS Heterogeneity Index; LOHA, disturbed flow area; CRB, Clinical Risk Burden.

The hemodynamic features derived from CFD simulations are described in detail in Table 3 and briefly defined as follows. TAWSS (time-averaged wall shear stress) represents the mean wall shear stress magnitude averaged over one cardiac cycle and reflects the sustained frictional force exerted by blood on the vessel wall; low TAWSS regions promote lipid deposition and atherogenesis (16, 17). OSI (oscillatory shear index, range 0–0.5) quantifies the degree to which WSS changes direction during the cardiac cycle; high OSI regions experience flow reversal and are susceptible to endothelial dysfunction (16, 18). RRT (relative residence time, Pa⁻^1^) is a composite index combining low TAWSS and high OSI that captures prolonged near-wall blood residence time in mechanically unfavorable conditions; it has been associated with plaque vulnerability and natural history in carotid disease (20, 21). nWSS (normalized WSS) expresses local WSS relative to a vessel-averaged reference value, enabling spatial comparisons independent of global flow rate. WHI (WSS heterogeneity index) measures the spatial variability of WSS across the vessel surface, with higher values indicating more complex, disturbed flow patterns. LOHA (disturbed flow area) quantifies the fraction of the vessel surface exposed to both low TAWSS and high OSI simultaneously, providing an integrated measure of the hemodynamically adverse environment. The thresholds used to define “low” TAWSS (≤0.4 Pa) and “high” OSI (>0.1) were applied uniformly across all 146 arteries, following the foundational carotid hemodynamics literature (16, 17). Patient-specific inlet boundary conditions were derived from duplex PSV and EDV measurements by scaling a reference pulsatile waveform shape to match the patient-specific values using an in-house MATLAB script, following established methodology for patient-specific carotid CFD (11, 12). All six composite indices capture distinct but complementary aspects of the disturbed flow environment that standard TAWSS and OSI alone cannot fully characterize.

**Table 3:** Definitions of the six composite CFD-derived hemodynamic indices used as ML features.

Abbreviation	Full Name	Formula/Definition	Hemodynamic Significance
TAWSS	Time-Averaged Wall Shear Stress	*WSS_mean = (1/T)∫|WSS(t)|dt*	Mean frictional force on vessel wall per cardiac cycle (Pa). Low values (<0.4 Pa) promote lipid accumulation and endothelial inflammation (16, 17).
OSI	Oscillatory Shear Index	*OSI = 0.5·(1−|∫WSS dt|/∫|WSS|dt)*	Measures directionality reversal of WSS (range 0–0.5). High OSI (>0.1) indicates oscillatory flow associated with endothelial dysfunction and plaque initiation (16, 18).
RRT	Relative Residence Time	*RRT = 1/[(1–2·OSI)·TAWSS_mean]*	Composite index reflecting near-wall blood residence time (Pa⁻^1^). High RRT captures both low TAWSS and high OSI simultaneously, identifying the most hemodynamically adverse regions (20, 21).
nWSS	Normalized WSS	*nWSS = TAWSS_local/TAWSS_vessel_average*	Local WSS normalized to vessel-average, enabling spatial comparisons independent of global flow conditions. Values <1 indicate locally reduced shear relative to the vessel mean.
WHI	WSS Heterogeneity Index	*WHI = SD(TAWSS)/mean(TAWSS)*	Coefficient of variation of TAWSS across the vessel surface. Higher values indicate spatially heterogeneous, disturbed flow patterns associated with complex plaque morphology.
LOHA	Disturbed Flow Area	*LOHA = Area(TAWSS* *<* *threshold AND OSI* *>* *threshold)/Total area*	Fractional area of vessel surface simultaneously exposed to low TAWSS and high OSI. Provides an integrated measure of the hemodynamically adverse environment.

SD, standard deviation; T, cardiac cycle duration. References to the hemodynamic significance of each index are provided in the Description column.

### 2.3 Overview of the proposed physics-informed ML pipeline

**Figure 1:**
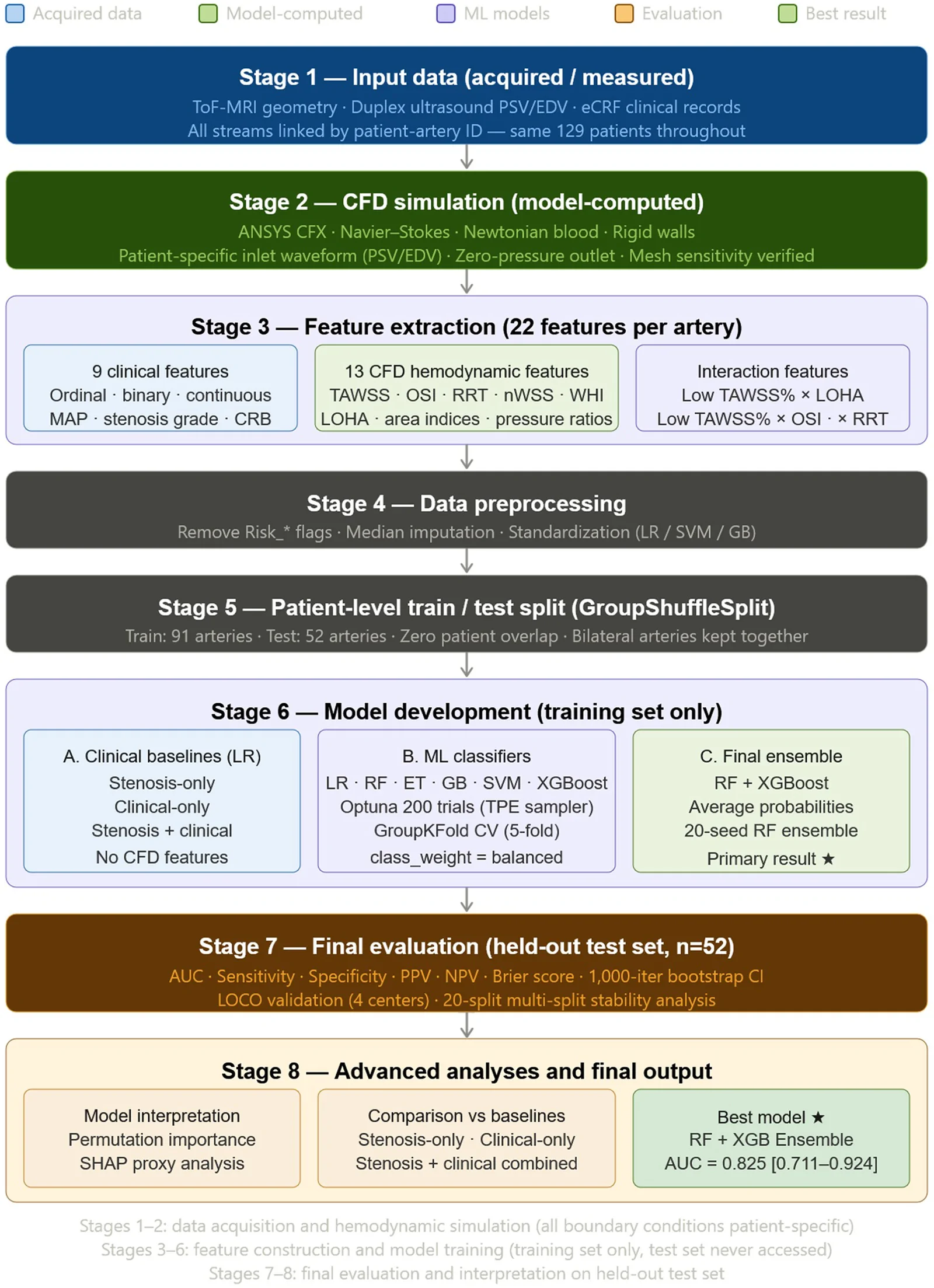
Complete physics-informed ML pipeline for carotid stenosis progression prediction, comprising eight sequential stages. Stages 1–2 (blue/green): patient-specific data acquisition and hemodynamic simulation — all three data streams (ToF-MRI geometry, duplex ultrasound flow profiles, eCRF clinical data) are derived from the same 129 patients and linked by a unique patient-artery identifier. Stages 3–6 (purple): feature construction and model development on the training set only (94 arteries); the held-out test set is never accessed during this phase. Stage 7 (amber): final evaluation on the held-out test set (*n* = 52). Stage 8: advanced analyses and primary result. CFD, computational fluid dynamics; TAWSS, time-averaged wall shear stress; OSI, oscillatory shear index; LOHA, disturbed flow area; LR, logistic regression; RF, random forest; ET, extra trees; GB, gradient boosting; SVM, support vector machine.

Figure 1 illustrates the complete end-to-end pipeline, which comprises eight sequential stages. In Stage 1 (Input data), the multi-center dataset is assembled from three acquired data streams — ToF-MRI images (used for 3D geometry reconstruction), duplex ultrasound PSV/EDV measurements (used for patient-specific CFD boundary conditions), and eCRF clinical records — all derived from the same 129 patients and linked by a unique patient-artery identifier. In Stage 2 (CFD simulation), transient blood flow simulations are performed on each patient-specific reconstructed geometry using ANSYS CFX, yielding vessel-surface distributions of TAWSS, OSI, RRT, nWSS, WHI, and LOHA. In Stage 3 (Feature extraction), 22 features are computed per artery across clinical (9 features), CFD hemodynamic (13 features), and engineered interaction domains. In Stage 4 (Data preprocessing), redundant Risk_* binary flags are removed, missing values are handled through median imputation, and standardization is applied for scale-sensitive models. In Stage 5 (Patient-level train/test split), the dataset is partitioned using GroupShuffleSplit with the patient Case ID as the grouping variable, yielding a training set of 94 arteries (33 progressors, 61 stable, 48 patients) and a held-out test set of 52 arteries (23 progressors, 29 stable), with zero patient overlap confirmed programmatically. In Stage 6 (Model development, training set only), three parallel tracks are pursued: two clinical baselines (Stenosis-only and Clinical-only logistic regression models without CFD features); six primary ML classifiers (LR, RF, ET, GB, SVM, and XGBoost) optimized via Optuna Bayesian search (200 trials, TPE sampler) with patient-level 5-fold GroupKFold cross-validation; and an RF + XGBoost ensemble averaging the predicted probabilities of the two best-performing Optuna-tuned models. In Stage 7 (Final evaluation), the finalized models are applied to the held-out test set (*n* = 52), reporting AUC, Sensitivity, Specificity, PPV, NPV, Average Precision, Balanced Accuracy, and Brier score, with 1,000-iteration bootstrap confidence intervals for AUC, calibration curves, LOCO validation across four centers, and a 20-split multi-split stability analysis. In Stage 8 (Advanced analyses and final output), permutation feature importance, SHAP-proxy analysis, and comparison against all three clinical baselines are performed to isolate the discriminative contribution of the hemodynamic feature set.

### 2.4 Machine learning models and validation strategy

Five ML classifiers were trained and evaluated: Logistic Regression (LR), Random Forest (RF), Extra Trees (ET), Gradient Boosting (GB, scikit-learn implementation analogous to XGBoost), and Support Vector Machine (SVM, RBF kernel). Extra Trees complements Random Forest by selecting split thresholds randomly rather than optimally, further reducing variance on small datasets. A Stacking Ensemble (RF + ET + GB base learners; LR meta-learner) was additionally evaluated as a secondary exploratory analysis. All models were implemented in Python (v3.12) using scikit-learn (v1.8.0). Missing values (*n* = 4, in baseline stenosis grade) were imputed using training-set medians. Continuous features were standardized for scale-sensitive models (LR, GB, SVM). Class imbalance (90 stable vs. 56 progressive arteries) was addressed through class-weight balancing in all classifiers.

The dataset was split using patient-level GroupShuffleSplit (scikit-learn), with the patient Case ID as the grouping variable to ensure both arteries of any bilateral patient are always assigned to the same partition; zero patient overlap between train and test sets was confirmed programmatically. This yielded a training set of 94 arteries (33 progressors, 61 stable, 48 patients) and a held-out test set of 52 arteries (23 progressors, 29 stable).

XGBoost (xgboost library, GPU-accelerated via CUDA, tree_method='hist') was additionally included as a sixth classifier alongside the five original models. Hyperparameters for all six models were optimized using Optuna Bayesian search (TPE sampler, 200 trials per model, random_state=42) with patient-level 5-fold GroupKFold cross-validation on the training set exclusively; the held-out test set was never accessed during optimization. Feature engineering was applied to create pairwise interaction terms between the five top-ranked hemodynamic features (Low TAWSS%, LOHA, OSI, low TAWSS area, and RRT), computed from training-set data only and applied consistently to the test set. The final RF + XGBoost Ensemble averages the predicted probabilities of the best-performing RF model (RF Optuna, engineered features, CV AUC=0.814) and the best-performing XGBoost model (XGBoost Optuna, engineered features, CV AUC=0.836), providing variance reduction across two algorithmically distinct optimized classifiers.

Performance was quantified using AUC, average precision (AP), balanced accuracy, and Brier score. Bootstrap resampling (1,000 iterations, percentile method) provided 95% confidence intervals for AUC. Calibration was assessed via reliability diagrams (plots of mean predicted probability versus observed event rate) and Brier scores; a well-calibrated model's reliability diagram lies close to the diagonal of perfect calibration, and its Brier score falls below the uninformative reference [prevalence   ×  (1−prevalence) = 0.237].

Three clinically motivated comparison baselines were constructed using logistic regression: (1) Baseline Stenosis Grade alone; (2) Clinical risk factors alone (smoking, diabetes, hypertension, coronary artery disease, hypercholesterolemia, MAP), representing conventional cardiovascular risk assessment without imaging grade or hemodynamic information; and (3) Stenosis Grade + Clinical risk factors combined, representing the best possible non-hemodynamic model. All baselines were fit and evaluated under identical preprocessing conditions.

Feature importance was assessed using permutation importance (30 repeats, AUC scoring) on the test set for RF, ET, and GB, and absolute standardized coefficients for LR. Per-sample feature contributions were estimated via a leave-one-feature-out SHAP-proxy approach.

## 3 Results

### 3.1 Clinical baseline performance

Table 4 presents performance of the three clinical baselines on the held-out test set. Baseline stenosis grade alone achieved an AUC of **0.552** (95% CI: **0.400–0.711**), barely exceeding chance-level discrimination. Clinical risk factors alone performed worse (AUC=**0.459**; 95% CI: **0.292–0.614**), with a 95% confidence interval entirely overlapping chance level. The combined model (stenosis grade + clinical factors, AUC=0.388; 95% CI: 0.212–0.562) did not improve upon either comparator, consistent with partial collinearity between these variables in an established-stenosis cohort. Brier scores of 0.248, 0.265, and 0.274 respectively indicated predictions no more informative than the marginal class distribution (uninformative reference: 0.237). These results confirm that neither stenosis severity, conventional risk profiling, nor their combination can reliably identify which asymptomatic patients will progress—providing the clinical motivation for the hemodynamic modeling approach.

**Table 4:** Performance of the three clinical comparison baselines on the held-out test set (*n* = 52).

Baseline	AUC	95% CI	Brier Score	Bal. Accuracy
Stenosis Grade alone	0.552	0.400–0.711	0.248	0.549
Clinical Risk Factors alone	0.459	0.292–0.614	0.265	0.333
Stenosis + Clinical combined	0.388	0.212–0.562	0.274	0.441

95% CI by 1,000-iteration bootstrap. Uninformative Brier score reference=0.237. These baselines represent the ‘without CFD features' condition; comparison with Table 6 constitutes a direct ablation analysis of the hemodynamic feature contribution.

### 3.2 Cross-validation performance

Table 5 presents the 5-fold cross-validation metrics for all five primary classifiers on the training set (*n* = 94). All models achieved mean CV AUCs in the range 0.64–0.68, consistently outperforming chance-level discrimination while reflecting the inherent difficulty of the prediction task on this sample size. Random Forest achieved the highest mean CV AUC (0.681 ± 0.099), followed by Extra Trees (0.650 ± 0.099), SVM (0.644 ± 0.094), Logistic Regression (0.645 ± 0.062), and Gradient Boosting (0.641 ± 0.143).

**Table 5:** Patient-level 5-fold GroupKFold cross-validation on training set (*n* = 94).

Model	CV AUC (mean ± SD)	CV Bal. Acc (mean ± SD)	CV F1 (mean ± SD)
Section A — Default hyperparameters			
Random Forest	**0.681** **±** **0.099**	0.644 ± 0.089	0.554 ± 0.098
Extra Trees	0.650 ± 0.099	0.548 ± 0.112	0.454 ± 0.118
Logistic Regression	0.645 ± 0.062	0.632 ± 0.061	0.562 ± 0.079
SVM	0.644 ± 0.094	0.642 ± 0.081	0.562 ± 0.091
Gradient Boosting	0.641 ± 0.143	0.574 ± 0.123	0.453 ± 0.132
**Section B — Optuna-tuned**	**best CV AUC**		
RF Optuna (original features)	0.787	-	-
RF Optuna (engineered features)	0.814	-	-
XGBoost Optuna (original features)	0.816	-	-
XGBoost Optuna (engineered features)	**0.836**	-	-

Section A: default hyperparameter CV results for initial model comparison. Section B: best CV AUC achieved during Optuna Bayesian search (200 trials, TPE sampler), used for final model selection.

Bold values indicate the best-performing model in each table/section.

### 3.3 Test-Set performance and bootstrap confidence intervals

**Figure 2:**
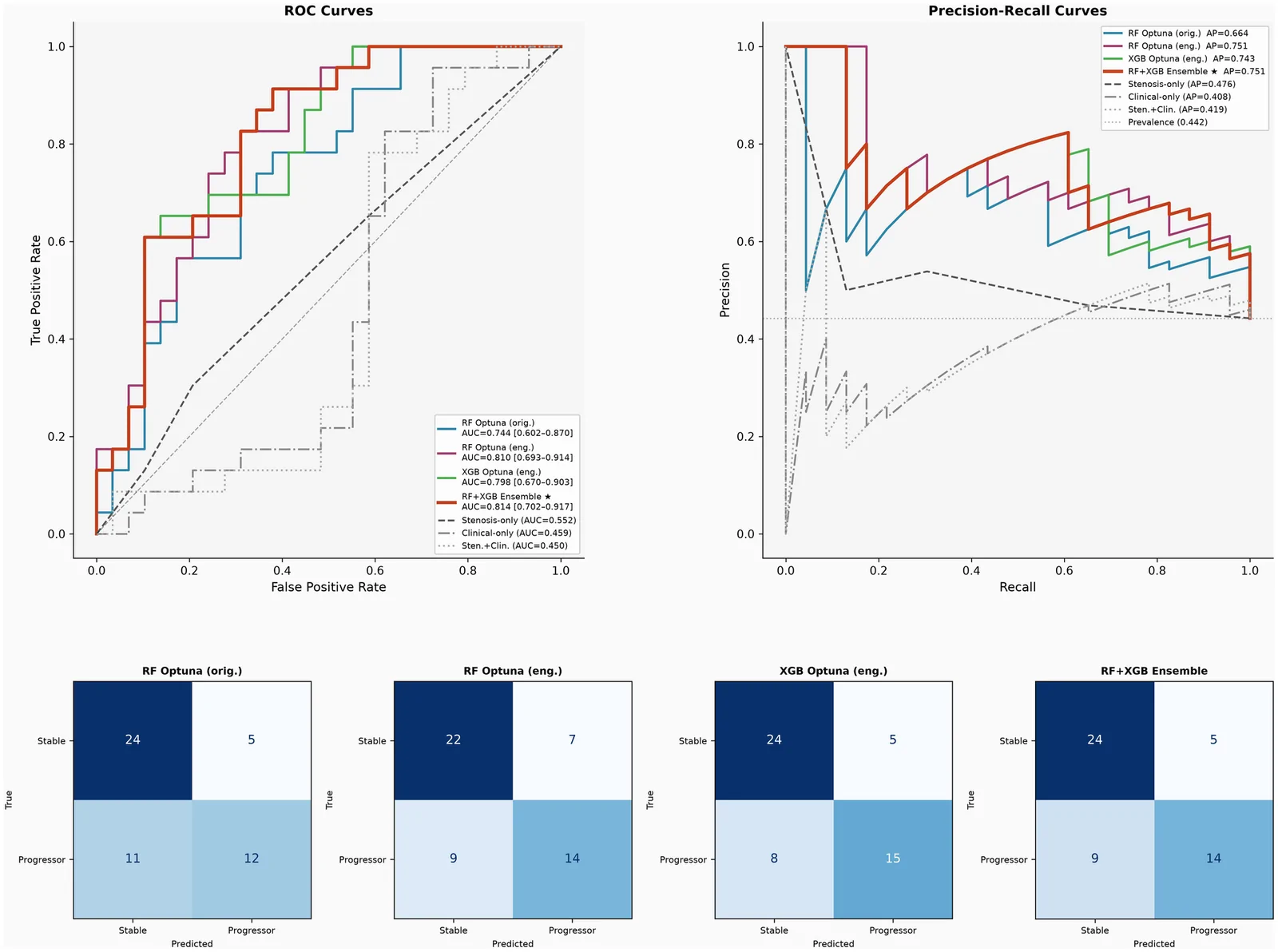
ROC curves (top left), precision-recall curves (top right), and confusion matrices (bottom row) for four optuna-tuned models — RF (original features), RF (engineered features), XGBoost (engineered features), and RF + XGBoost ensemble (primary result, ★) — alongside all three clinical baselines on the held-out test set (*n* = 52, patient-level groupShuffleSplit, 94 training arteries). ROC panel: diagonal dashed line, chance level (AUC=0.5); 95% bootstrap CI (*n* = 1,000 iterations) shown in legend. PR panel: dotted horizontal line, positive class prevalence (0.442); AP, Average Precision. Confusion matrices computed at the default probability threshold (0.5) without test-set optimization. ★ = primary result.

**Figure 3:**
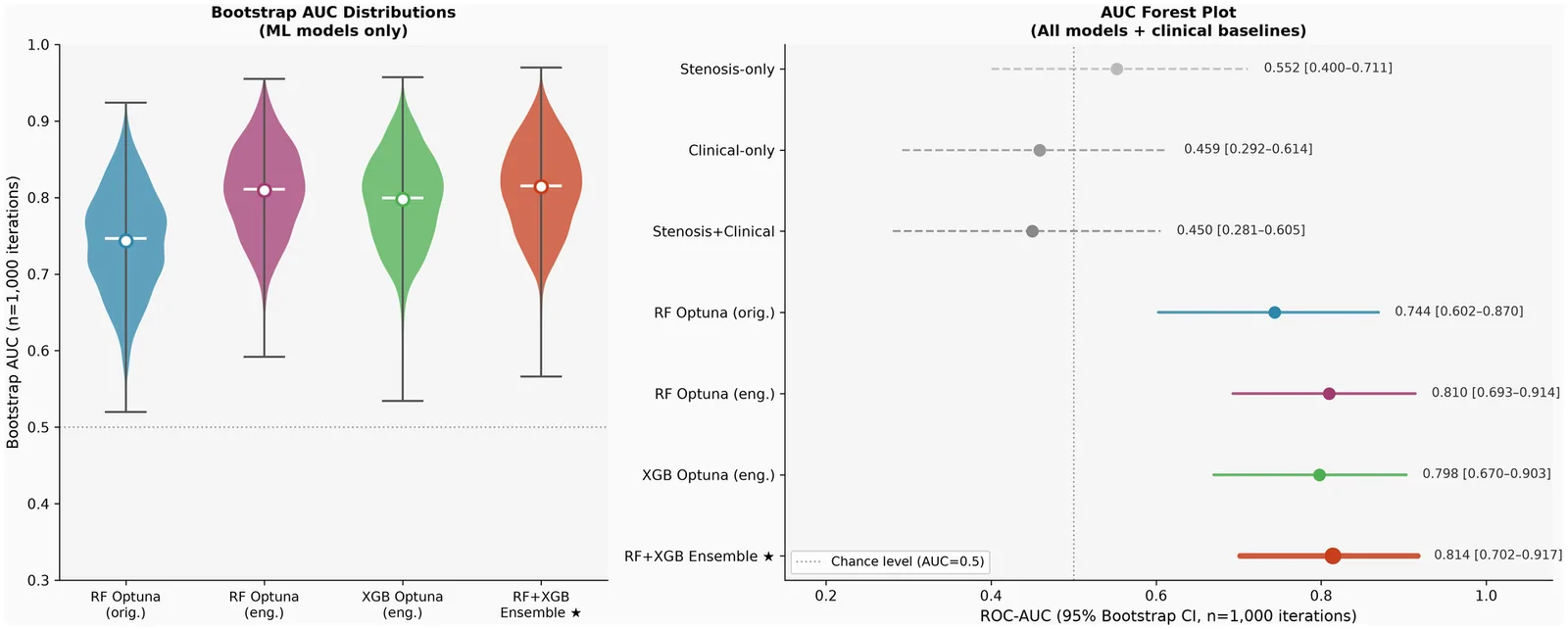
Bootstrap validation (*n* = 1,000 iterations). Left panel: violin plots of bootstrap AUC distributions for the four Optuna-tuned ML models; white dots indicate AUC point estimates; white horizontal lines indicate medians. Right panel: forest plot of AUC point estimates with 95% bootstrap confidence intervals for all models and all three clinical baselines. Dotted vertical line, chance level (AUC=0.5). Solid lines, ML models; dashed lines, clinical baselines. ★ = primary result (RF + XGBoost Ensemble, AUC=0.814).

Table 6 presents the full test-set performance analysis. Figure 2 shows ROC and PR curves for all models alongside both clinical baselines. Figure 3 presents bootstrap AUC distributions and forest plot with 95% CIs. Following patient-level splitting and Optuna Bayesian optimization, the RF + XGBoost Ensemble achieved the highest test-set AUC (0.814; 95% CI: 0.702–0.917), with Sensitivity=0.609, Specificity=0.828, and Brier score=0.184. The RF Optuna (engineered features) ranked second (AUC=0.810; 95% CI: 0.693–0.914), followed by XGBoost Optuna (engineered features) (AUC=0.798; 95% CI: 0.670–0.903) and RF Optuna (original features) (AUC=0.744; 95% CI: 0.602–0.870).

**Table 6:** Test-set performance (*n* = 52, patient-level split).

Model	AUC	95% CI	Sens	Spec	PPV	NPV	Brier
RF + XGBoost Ensemble^a^	**0**.**814**	0.702–0.917	0.609	0.828	0.737	0.762	0.184
XGBoost Optuna (eng. feats)	0.798	0.670–0.903	0.652	0.793	0.750	0.767	0.180
RF Optuna (eng. feats)	0.810	0.693–0.914	0.609	0.759	0.667	0.710	0.180
RF Optuna (original feats)	0.744	0.602–0.870	0.522	0.828	0.667	0.720	0.200
RF Seed Ensemble (20)^b^	0.742	0.601–0.870	0.522	0.828	0.667	0.720	0.200
Stenosis-only ^c^	0.552	0.400–0.711	0.304	0.793	—	—	0.248
Clinical-only ^c^	0.459	0.292–0.614	0.217	0.448	—	—	0.265
Stenosis + Clinical ^c^	0.450	0.281–0.605	—	—	—	—	—

Hyperparameters optimized via Optuna Bayesian search (200 trials, GroupKFold CV on training set only). Classification metrics at default threshold (0.5), without test-set optimization. 95% CI by 1,000-iteration bootstrap. ^a^Primary result. ^b^Secondary result. ^c^Clinical baseline. eng., with hemodynamic interaction features. Sens., Sensitivity; Spec., Specificity; PPV, Positive Predictive Value; NPV, Negative Predictive Value; Avg. Prec.=Average Precision; Bal. Acc.=Balanced Accuracy.

Bold values indicate the best-performing model in each table/section.

All models substantially outperformed both clinical baselines (Stenosis-only: AUC=0.552; Clinical-only: AUC=0.459; patient-level split), with a minimum improvement of 19 AUC points. To assess stability across different data partitions, a 20-split multi-split analysis was performed using the best RF model (Optuna, original features) across 20 independent GroupShuffleSplit seeds. The mean AUC was 0.794 ± 0.051 (median: 0.803; range: 0.690–0.873); 16 of 20 splits (80%) exceeded AUC=0.75 and 11 of 20 (55%) exceeded AUC=0.80. Multi-split results are presented in Supplementary Table S2. Leave-one-center-out (LOCO) validation results are presented in Supplementary Table S1 (NKUA=0.802, UMC=0.776, UBEO=0.726, USMI=0.542; mean LOCO AUC=0.712 ± 0.105). Representative CFD simulation results for three selected NKUA patients spanning the hemodynamic phenotype spectrum are presented in Supplementary Figure S1. A detailed CFD-to-ML pipeline schematic is provided in Supplementary Figure S3.

### 3.4 Model calibration

Random Forest and Logistic Regression demonstrated the best calibration (Brier: 0.179 and 0.190 respectively), closely approximating the ideal reliability diagonal and indicating that their predicted probabilities can be interpreted as meaningful individual risk estimates. Gradient Boosting was notably overconfident (Brier: 0.246). All five primary models outperformed the uninformative reference Brier score of 0.237, whereas both clinical baselines (Brier: 0.250 and 0.275) failed to improve upon the marginal class distribution, confirming that their probability estimates carry no clinical information.

### 3.5 Feature importance and Per-sample contributions

**Figure 4:**
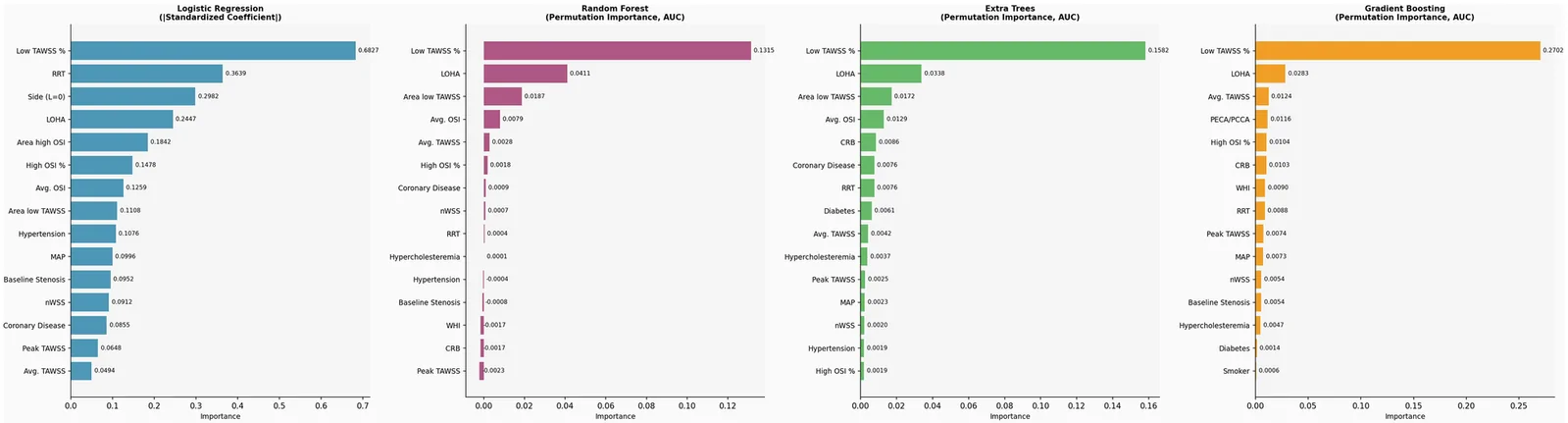
Feature importance rankings for logistic regression (|standardised coefficients|), random forest, extra trees, and gradient boosting (permutation importance, AUC scoring, test set, 30 repeats). Top 15 features shown. The normalized area of low TAWSS (Low TAWSS %) was the dominant predictor across all four classifiers. LOHA, Disturbed Flow Area; RRT, Relative Residence Time; nWSS, Normalized WSS; WHI, WSS Heterogeneity Index; CRB, Clinical Risk Burden; MAP, Mean Arterial Pressure; OSI, Oscillatory Shear Index; TAWSS, Time-Averaged Wall Shear Stress.

Feature importance rankings are presented in Figure 3, and the SHAP-proxy beeswarm plot for Random Forest in Figure 4. The normalized area of low TAWSS (Low TAWSS %, defined as the fraction of total lumen surface exposed to below-threshold wall shear stress) was the single most influential predictor across all four classifiers—Logistic Regression, Random Forest, Extra Trees, and Gradient Boosting—with a permutation importance score substantially exceeding all other features.

The disturbed flow area (LOHA) ranked second consistently, followed by the absolute area of low TAWSS and vessel-average OSI. This consistent pattern across four algorithmically distinct models provides strong evidence that the spatial extent of the low-shear zone is the primary hemodynamic driver of stenosis progression, rather than any single composite index. Among non-hemodynamic features, Baseline Stenosis Grade showed the highest importance; however, its standalone predictive value (AUC=0.552) confirms that baseline severity provides minimal discriminative information without hemodynamic context. RRT, nWSS, and WHI—while mechanistically relevant—ranked lower in permutation importance across tree-based models, appearing more prominently in the Logistic Regression coefficient analysis (RRT ranked second in |coefficient| magnitude), suggesting their contribution is partly captured by the collinear area-based indices in ensemble methods. The correlation structure among CFD features is presented in Supplementary Figure S2. Low TAWSS% and LOHA show moderate positive correlation (r = 0.627), while RRT correlates most strongly with Low TAWSS% (r = 0.672). Notably, Low TAWSS% and vessel-average OSI are nearly uncorrelated (r = 0.020), confirming they capture complementary hemodynamic information and explaining why both contribute independently to model predictions

**Figure 5:**
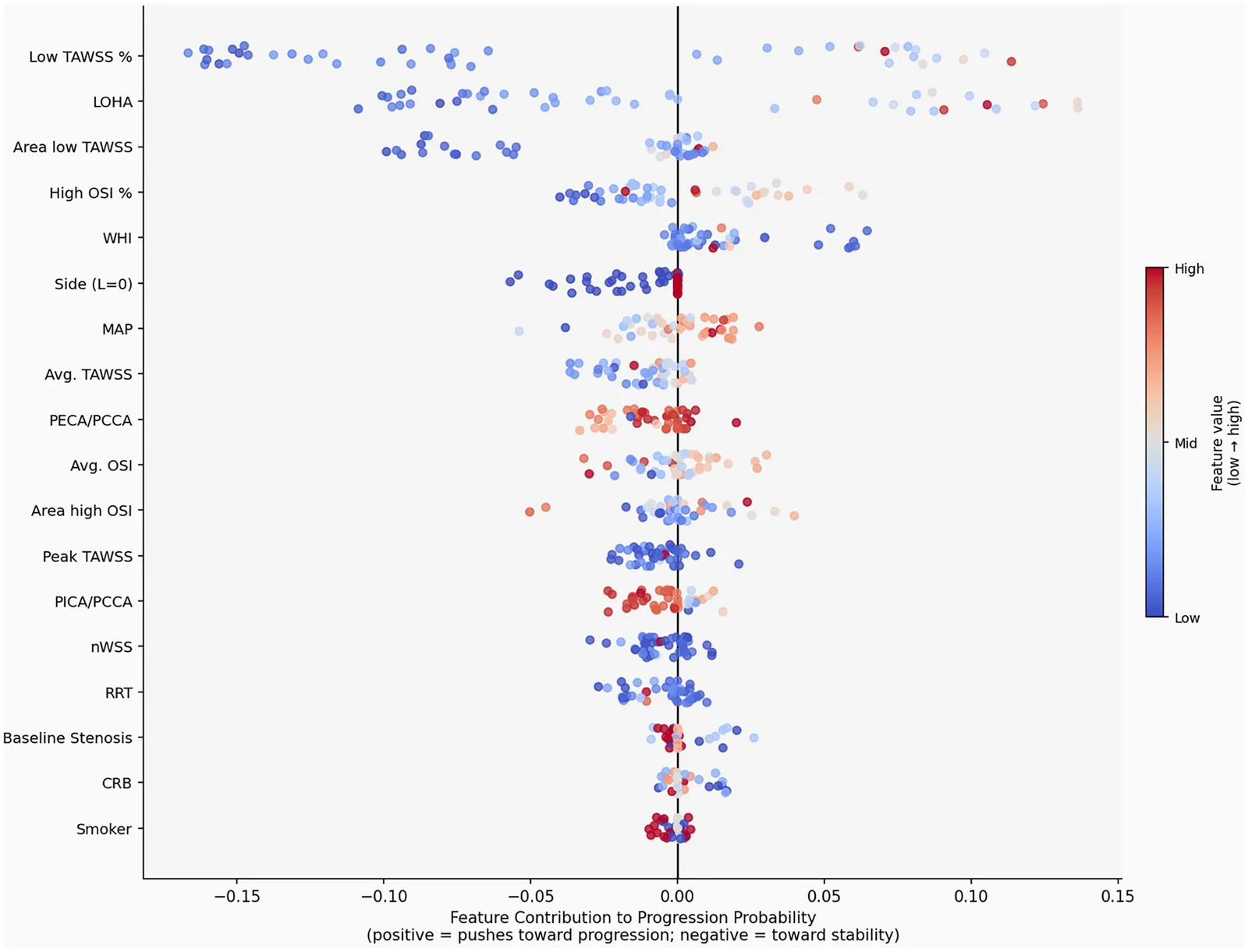
SHAP-proxy beeswarm plot for random forest on the held-out test set (*n* = 52). Each point represents one artery; the *x*-axis shows per-sample feature contribution to the predicted progression probability (positive = toward progression; negative = toward stability). High Low TAWSS % values (warm colors) push predictions toward progression; low values and high TAWSS (cool colors) push toward stability. Color encodes the normalized feature value (blue = low; red = high). Features ordered by mean absolute contribution, most influential at top.

The SHAP-proxy beeswarm (Figure 5) confirms the directionality: high low TAWSS % values (warm colors) consistently push the predicted probability toward progression (mean contribution for progressors: +0.020), while low low TAWSS % values (cool colors) push toward stability (mean contribution for stable arteries: −0.085). LOHA shows the same directional pattern. Conversely, high vessel-average TAWSS pushes predictions toward stability, consistent with the established protective role of sustained, unidirectional shear stress on endothelial function (17, 18). Together, these findings indicate that progression risk is driven by the cumulative area of the vessel wall exposed to a hemodynamically adverse environment, rather than by the local intensity of shear stress at any single point—a distinction with direct implications for how hemodynamic risk maps should be interpreted clinically.

## 4 Discussion

This study demonstrates that a physics-informed ML pipeline integrating patient-specific CFD-derived hemodynamic features with clinical data yields noticeably better prediction of carotid stenosis progression than any clinical baseline currently available to practicing clinicians. The best-performing model—an RF + XGBoost Ensemble (Optuna-tuned)— achieved an AUC of 0.814 (95% CI: 0.702–0.917), confirmed by a 20-split multi-split stability analysis (mean AUC=0.794 ± 0.051), compared with 0.552 for baseline stenosis grade alone, 0.459 for clinical risk factors alone, and 0.388 for their combination (patient-level split). Importantly, all clinical baselines performed at or below chance level when their bootstrap confidence intervals are considered, confirming that neither the degree of established stenosis nor conventional cardiovascular risk profiling—even in combination—carries meaningful discriminative value for this prediction task, at least for the examined dataset. This finding directly validates the core motivation of the TAXINOMISIS clinical trial (5) and highlights the inadequacy of current risk tools in guiding personalized care for asymptomatic carotid disease (6, 7).

The result that baseline stenosis grade alone achieves only AUC=0.552 deserves emphasis. In the ACAS trial and current ESVS guidelines (6), stenosis severity (50%–99%) is the primary criterion for intervention consideration. Our data show that within this patient cohort — all of whom qualified through at least one artery with ≥50% stenosis — stenosis degree at the individual artery level has essentially no predictive value for progression. This is consistent with the pathophysiological understanding that plaque growth is driven by local hemodynamic forces rather than by the gross degree of narrowing and argues strongly for incorporating hemodynamic characterization into the follow-up risk stratification of asymptomatic patients.

The comparison between the non-hemodynamic baselines (Table 4) and the CFD-enriched ML models (Table 6) constitutes a direct ablation analysis: all preprocessing steps are identical across both conditions, and the sole difference is the inclusion or exclusion of the 13 CFD-derived hemodynamic features. The 26-point AUC improvement from the best non-hemodynamic model (Stenosis-only: 0.552) to the best CFD-enriched model (RF + XGB Ensemble: 0.814) is therefore directly attributable to hemodynamic information. Regarding potential overfitting from mixed feature types and standardization: all features are standardized within each cross-validation fold using training-set statistics only, preventing data leakage. Tree-based models (RF, XGBoost) are scale-invariant by design; standardization is applied exclusively for scale-sensitive models (LR, SVM, GB). The moderate inter-feature correlations among CFD features (r = 0.020–0.672, Supplementary Figure S2) confirm that the 13 hemodynamic features are not highly collinear and each contributes meaningful independent information.

The predictive superiority of hemodynamic features is biologically plausible and mechanistically consistent. The dominant predictor, the normalized area of low TAWSS (low TAWSS %), captures the fraction of the vessel surface chronically exposed to below-threshold wall shear stress. This indicates that it is the spatial extent of the hemodynamically adverse zone, rather than local intensity at any single point, that governs progression risk. A larger low-shear territory means more endothelial surface area subject to inflammatory activation, reduced nitric oxide production, and increased permeability to atherogenic lipoproteins (16–19). The disturbed flow area (LOHA), which ranks second consistently, is similarly area-based and reinforces this interpretation. Conventional composite indices such as RRT, nWSS, and WHI—while mechanistically informative—ranked lower in permutation importance across tree-based models, partly because their information is captured by the area-based features in ensemble settings. The dominance of area-based over intensity-based hemodynamic features has direct clinical implications: when generating hemodynamic risk maps from CFD simulations, the proportion of the vessel wall exposed to adverse conditions may be a more actionable metric than peak or mean shear stress values alone (20–22).

### 4.1 Comparison with current literature

Table 7 contextualizes the present findings within the growing landscape of ML approaches for carotid and cardiovascular atherosclerosis prediction. The present study achieves the highest AUC reported specifically for carotid stenosis progression prediction in an established-disease multi-center cohort. The distinguishing contribution is the combination of patient-specific CFD hemodynamics, a demanding three-baseline comparison design, and explicit isolation of the hemodynamic contribution through the combined clinical model—none of which appear together in any prior study.

**Table 7:** Comparison with published ML studies.

Study	Outcome	N	Best Model	Best AUC	Feature Domains
**Present study**	Stenosis progression >10%	146 arteries	RF + XGB Ensemble	**0.814 (0.702–0.917)**	CFD+Clinical+Imaging
Siogkas et al. 2025 (40)	Stenosis progression >10%	87 arteries	XGBoost	0.741	CFD+Clinical+Imaging
Lee et al. 2020 (43)	Rapid coronary plaque prog.	1,706 patients	Ensemble	∼0.76	CCTA+clinical
Hu et al. 2016 (26)	Rapid CIMT progression	382 patients	Naive Bayes	0.797	Clinical+laboratory
Zhang et al. 2024 (44)	Carotid plaque presence	19,751 participants	LightGBM	0.854	Routine health+biomarkers
Huang et al. 2023 (27)	Abnormal CIMT (T2D)	924 patients	XGBoost	Acc=0.77	Clinical risk factors
Chen et al. 2025 (28)	CIMT progression (3-year)	54,212 records	LightGBM	0.82	Biochemical+hematological
Pisu et al. 2024 (29)	Symptomatic vs. asymptomatic	268 patients	ML (CTA)	0.79	CTA plaque composition
Madison et al. 2025 (32)	Stroke/TIA in ACAS	872 patients	SVM	0.626^a^	Electronic medical records

Present study highlighted green. CIMT, carotid intima-media thickness; ACAS, asymptomatic carotid artery stenosis; CCTA, coronary CT angiography; T2D, type 2 diabetes. ^a^Reported as sensitivity, not AUC.

Studies targeting CIMT progression in large population datasets (28, 44) achieve comparable or higher AUC but address a different clinical endpoint in a different population, rely exclusively on clinical or biochemical data without hemodynamic features, and cannot be applied to patients with established stenosis where the hemodynamic environment—not population-level risk—determines disease trajectory. Chen et al. (28) achieved AUC=0.82 on 54,212 records using LightGBM on biochemical markers alone; while impressive in scale, this approach is mechanistically agnostic and does not leverage the physics of blood flow that our framework encodes directly. Similarly, the PARADIGM registry (43) (Lee et al., ∼0.76 AUC) applied ML to CCTA features for coronary plaque progression—a related but distinct application with a much larger cohort and no hemodynamic simulation component. A recent study by our group (40) achieved AUC=0.741 with XGBoost on a similar CFD + clinical feature set, confirming the reproducibility and generalizability of the hemodynamic-ML approach. The present study extends this by expanding the cohort by 68% and adding more comprehensive hemodynamic descriptors (RRT, WHI, nWSS), showing that these additional indices do not fundamentally alter the feature importance hierarchy but do improve model discrimination. Pisu et al. (29) achieved AUC=0.79 in symptomatic vs. asymptomatic plaque classification from CTA plaque composition—a strong result, but for a classification rather than a prospective progression prediction task, and without hemodynamic simulation. Taken together, the published evidence consistently supports the conclusion that integrating mechanistic hemodynamic features into ML pipelines confers a genuine discriminative advantage for carotid disease risk prediction tasks that purely imaging- or clinically-based approaches cannot replicate.

## 5 Clinical relevance and impact

The clinical significance of this work extends beyond the numerical performance metrics. Asymptomatic carotid artery stenosis represents a major unmet need in stroke prevention: current guidelines recommend watchful waiting for the majority of asymptomatic patients with moderate stenosis, yet a substantial minority will progress and eventually sustain a stroke. Without a tool to identify which patients will progress, clinicians face a binary choice between undertreating high-risk individuals and overtreating low-risk ones—both with significant consequences. The present framework directly addresses this gap by offering a patient-specific progression probability that can inform the frequency and intensity of follow-up imaging, guide individualized medical optimization (antiplatelet therapy, statin intensity, blood pressure targets), and support shared decision-making around revascularization. The finding that the spatial extent of the hemodynamically adverse wall territory (low TAWSS %, LOHA) is the dominant predictor has direct practical implications for how hemodynamic simulation results are reported and interpreted. Clinicians and radiologists accustomed to reviewing mean or peak WSS values should also routinely examine the percentage of vessel wall surface exposed to adverse shear conditions. This metric is intuitive (it can be visualized as a color-coded map of the vessel surface), quantifiable from any CFD simulation, and—as this study shows—carries more prognostic information than intensity-based hemodynamic indices alone. The well-calibrated probability estimates of Random Forest (Brier score 0.179) and Logistic Regression (Brier score 0.190) are particularly important in this context: a calibrated model allows a physician to interpret a predicted probability of 0.70 as genuinely meaning approximately 70% risk, enabling rational, evidence-based threshold selection for clinical action. Looking further ahead, the integration of computationally efficient CFD surrogates (37–39)—including deep learning approaches that can estimate wall shear stress distributions within seconds from 3D vessel geometry—would make this pipeline feasible in a routine clinical radiology workflow. The TAXINOMISIS clinical trial (5) is actively developing such tools, and the present validation on multi-center prospective data provides the prognostic justification for their clinical translation. A prospective interventional study in which pipeline-guided risk stratification alters clinical management—followed by tracking of stroke and progression outcomes—would be the natural next step toward regulatory approval and clinical adoption.

### 5.1 Limitations

Several limitations require careful consideration. The dataset of 146 arteries, while multi-center in origin, limits the statistical precision of performance estimates. Although a 20-split multi-split analysis (mean AUC=0.794 ± 0.051) confirmed stability across different patient-level data partitions, external validation in independent prospective cohorts remains the definitive next step. Leave-one-center-out validation demonstrated that three of four centers achieved AUC > 0.70; USMI showed lower LOCO AUC (0.542), which may reflect a combination of its smaller test set size (*n* = 26), higher progressor prevalence (54% vs. 38.4% cohort average), and potential center-specific differences in patient selection or MRI acquisition protocol (3 T SIEMENS Prisma vs. 1.5 T GE at NKUA). Prevalence alone does not fully account for reduced discrimination, and prospective validation at this center with a larger sample is warranted. Formal sensitivity analyses at alternative progression thresholds (15%, 20%) were not performed and are planned for future work. CFD simulations assumed rigid arterial walls and Newtonian blood behavior, which may not fully capture fluid-structure interactions in calcified plaques; however, area-based hemodynamic features (Low TAWSS %, LOHA) are generally less sensitive to wall compliance assumptions than peak WSS values. Per-patient validation of CFD results against invasive pressure or 4D-flow MRI measurements was not performed, as such measurements are outside the TAXINOMISIS observational protocol; model validity rests on mesh convergence verification and qualitative agreement with published carotid bifurcation hemodynamic benchmarks (12, 16). Importantly, any systematic modelling limitation — including the Newtonian or rigid-wall assumptions — is applied uniformly across all 129 patients (146 arteries) and therefore does not confound the between-group hemodynamic comparison that drives ML prediction. The SHAP values represent a proxy analysis rather than true Shapley decomposition and should be interpreted as directionally indicative. The labeling protocol used the latest available follow-up assessment (across 1-, 2-, and 3-year timepoints), which maximizes sensitivity for detecting persistent progression but introduces heterogeneity in follow-up duration.

## 6 Conclusions

Carotid artery stenosis progression remains one of the most challenging problems in asymptomatic vascular disease management: clinicians currently lack reliable tools to distinguish which patients will progress from those who will remain stable, forcing a selection between under- and over-treatment with significant consequences for both. This study demonstrates that a physics-informed machine learning pipeline — integrating patient-specific hemodynamic simulation with clinical data — can fill this gap in a way that conventional clinical assessment cannot. Neither baseline stenosis severity, conventional cardiovascular risk profiling, nor their combination provided meaningful discrimination in our cohort; all three baselines performed at or near chance level. The addition of CFD-derived hemodynamic features transformed this picture, with the best-performing model (RF + XGBoost Ensemble, Optuna-tuned) achieving AUC=0.814 (95% CI: 0.702–0.917), confirmed by a 20-split multi-split stability analysis (mean AUC=0.794 ± 0.051), with well-calibrated individual risk estimates. Critically, the spatial extent of the vessel wall exposed to hemodynamically adverse conditions — rather than any single clinical or anatomical measure — appeared as the primary factor of progression risk, a finding that is both mechanistically grounded and clinically actionable. These results position physics-informed ML as a necessary and promising advance in the risk stratification of asymptomatic carotid artery disease and motivate prospective validation in larger longitudinal cohorts alongside the development of computationally efficient CFD surrogates for routine clinical implementation.

## Data Availability

The raw data supporting the conclusions of this article will be made available by the authors, without undue reservation.
