# Supplementary material for: Risk stratification in intermediate asymptomatic carotid artery disease: a multi-modal approach combining local flow dynamics and systemic clinical profiles
Source: Front Surg. 2026 Aug 28;13:1872735. doi: 10.3389/fsurg.2026.1872735 (PMC13562003; doi:10.3389/fsurg.2026.1872735)
Supplement: Supplementary file 1 [file Supplementaryfile1.docx]

**Supplementary Material**

**Supplementary Tables**

Supplementary Table S1: Leave-one-center-out (LOCO) validation results for the Optuna-tuned Random Forest. For each center, the model was trained on the remaining three centers and evaluated on the held-out center. 95% CI by 500-iteration bootstrap. The variation in sensitivity/specificity at the default 0.5 threshold reflects differences in center-specific progressor prevalence rather than a failure of model discrimination, as confirmed by the per-center AUC values.

| **Center** | **Train n** | **Test n** | **Progressors** | **AUC** | **95% CI** | **Bal. Acc.** | **Sensitivity** | **Specificity** |
| --- | --- | --- | --- | --- | --- | --- | --- | --- |
| NKUA | 99 | 47 | 12 (25.5%) | 0.802 | 0.657–0.934 | 0.638 | 0.333 | 0.943 |
| UBEO | 109 | 37 | 16 (43.2%) | 0.726 | 0.548–0.874 | 0.564 | 0.938 | 0.190 |
| UMC | 110 | 36 | 14 (38.9%) | 0.776 | 0.629–0.906 | 0.714 | 0.929 | 0.500 |
| USMI | 120 | 26 | 14 (53.8%) | 0.542 | 0.300–0.789 | 0.548 | 0.429 | 0.667 |
| **Mean** | — | — | — | **0.712 ± 0.105** | — | 0.616 | 0.657 | 0.575 |

*Bal. Acc.=Balanced Accuracy. Yellow row (USMI): lower AUC attributable to higher progressor prevalence (53.8%) when held out. Green row: mean across all four centers.*

Supplementary Table S2: Multi-split AUC stability analysis. The Optuna-tuned Random Forest (original features) was evaluated across 20 independent patient-level GroupShuffleSplit random seeds. Each seed produces a different train/test partition while maintaining patient-level integrity (bilateral arteries always assigned to the same partition). Mean AUC=0.794 ± 0.051; Median=0.803; Range=0.690–0.873.

| **Seed** | **AUC** | **Test n** | **Progressors in test** |
| --- | --- | --- | --- |
| 0 | 0.728 | 43 | 12 |
| 1 | 0.746 | 46 | 18 |
| 2 | 0.818 | 41 | 16 |
| 3 | 0.751 | 42 | 15 |
| 4 | 0.804 | 54 | 21 |
| 5 | 0.826 | 42 | 18 |
| 6 | 0.806 | 46 | 15 |
| 7 | 0.755 | 47 | 17 |
| 8 | 0.843 | 38 | 13 |
| 9 | 0.799 | 52 | 21 |
| 10 | 0.839 | 41 | 14 |
| 11 | 0.799 | 41 | 14 |
| 12 | 0.697 | 51 | 20 |
| 13 | 0.850 | 47 | 15 |
| 14 | 0.873 | 46 | 18 |
| 15 | 0.802 | 42 | 15 |
| 16 | 0.774 | 45 | 16 |
| 17 | 0.838 | 32 | 13 |
| 18 | 0.690 | 41 | 13 |
| 19 | 0.846 | 46 | 19 |
| **Mean ± SD** | **0.794 ± 0.051** | 44.0 | 16.2 |
| **Median** | **0.803** | — | — |
| **Range** | **0.690–0.873** | — | — |

*Yellow rows: two lowest AUC splits (seeds 12 and 18), both corresponding to test sets with higher-than-average progressor counts. Green rows: summary statistics. 16/20 splits (80%) exceeded AUC=0.75; 11/20 splits (55%) exceeded AUC=0.80.*

Supplementary Table S3: CFD-derived hemodynamic features: mean ± SD for stable arteries (Class 0, n=90) and progressor arteries (Class 1, n=56). Features with notable between-group differences are highlighted in green — these correspond to the top-ranked predictors in the feature importance analysis. TAWSS=time-averaged wall shear stress; OSI=oscillatory shear index; RRT=relative residence time; nWSS=normalized WSS; WHI=WSS heterogeneity index; LOHA=disturbed flow area; PICA=internal carotid artery pressure; PECA=external carotid artery pressure; PCCA=common carotid artery pressure.

| **Feature** | **Unit** | **Stable (n=90) mean ± SD** | **Progressors (n=56) mean ± SD** |
| --- | --- | --- | --- |
| Peak TAWSS | Pa | 150.39 ± 211.09 | 95.10 ± 113.44 |
| Vessel-average TAWSS | Pa | 10.83 ± 7.26 | 8.40 ± 4.88 |
| Vessel-average OSI | 0–0.5 | 0.175 ± 0.042 | 0.186 ± 0.039 |
| Area of low TAWSS | m² | 0.0001 ± 0.0001 | **0.0002 ± 0.0001** |
| Low TAWSS area / Total area (%) | % | 17.11 ± 12.01 | **33.57 ± 18.33** |
| Area of high OSI | m² | 0.0002 ± 0.0002 | 0.0002 ± 0.0001 |
| High OSI area / Total area (%) | % | 22.32 ± 25.96 | 21.00 ± 7.85 |
| PICA / PCCA | ratio | 0.934 ± 0.085 | 0.957 ± 0.039 |
| PECA / PCCA | ratio | 0.938 ± 0.075 | 0.938 ± 0.085 |
| RRT | Pa⁻¹ | 0.207 ± 0.137 | 0.275 ± 0.229 |
| nWSS | dimensionless | 0.0123 ± 0.0184 | 0.0074 ± 0.0087 |
| WHI | dimensionless | 13.10 ± 14.35 | 12.61 ± 14.52 |
| LOHA | dimensionless | 39.43 ± 28.97 | **54.58 ± 19.92** |

*Green rows indicate features with the largest between-group differences, corresponding to the dominant predictors in feature importance analysis (Low TAWSS area %, LOHA). All values computed from patient-specific CFD simulations. Class 0=stable; Class 1=progressor (>10% NASCET stenosis increase at latest follow-up).*

**Supplementary Figures**

Supplementary Figure S1. Representative CFD simulation results for three NKUA patients spanning the hemodynamic phenotype spectrum: A) low-risk stable artery (Case ID 14, Right, LOHA=7.13, Low TAWSS%=4.42%), B) intermediate progressor (Case ID 9, Left, LOHA=48.89, Low TAWSS%=27.70%), and C) high-risk progressor (Case ID 45, Right, LOHA=60.46, Low TAWSS%=40.50%). For each patient, four hemodynamic maps are shown on the reconstructed 3D carotid geometry: velocity streamlines, TAWSS distribution, OSI distribution, and RRT distribution. Color scales are consistent across patients to enable direct visual comparison.

**Supplementary Figure S2.** Pearson correlation matrix of all 13 CFD-derived hemodynamic features across the 146-artery cohort. Values shown in each cell. Color scale: red=strong positive correlation, blue=strong negative correlation, white=no correlation.

**Supplementary Figure S3.** Detailed CFD-to-ML pipeline schematic showing the full computational chain from patient geometry reconstruction through hemodynamic simulation to ML feature matrix assembly and model training and validation.
